# Extracellular Vesicles Inhibit Proliferation and Invasion of Ovarian Endometrial Stromal Cells and Their Expression of SF-1, ERβ, and Aromatase

**DOI:** 10.3389/fendo.2021.666195

**Published:** 2021-08-31

**Authors:** XiaoQing Wang, PeiLi Wu, Xin Li, Cheng Zeng, JingWen Zhu, YingFang Zhou, Ye Lu, Qing Xue

**Affiliations:** Department of Obstetrics and Gynecology, Peking University First Hospital, Beijing, China

**Keywords:** endometriosis, human umbilical cord mesenchymal stem cells, EVs, steroidogenic factor-1, ERβ, aromatase, proliferation, invasion

## Abstract

**Objective:**

Endometriosis is an estrogen-dependent chronic disease. The abnormal proliferation and invasion of ectopic stromal cells (ESCs) are important manifestations of endometriosis, and it is necessary to find safer and more effective treatments. Extracellular vesicles (EVs) derived from human umbilical cord mesenchymal stem cells (UC-MSCs) have been shown to be promising for the treatment of many diseases, except endometriosis. The main purpose of this study was to explore the effect of EVs derived from UC-MSCs on ESCs and evaluate the therapeutic value of EVs on endometriosis.

**Study Design:**

Following the successful culture and identification of UC-MSCs, we collected the medium of UC-MSCs and extracted EVs by ultracentrifugation. Then, 120 μg/mL EVs were used to stimulate ESCs, which were collected to evaluate cell proliferation and invasion and expression of the estrogen-related proteins steroidogenic factor-1 (SF-1), estrogen receptors β (ERβ), and aromatase.

**Results:**

Compared with the control group treated with isodose phosphate buffered saline (PBS), 120 μg/mL EVs exposure significantly decreased the expression of cyclin D1 (mRNA: n = 6, P = 0.02; protein: n = 6, P = 0.000) and matrix metalloproteinase (MMP) 9 (mRNA: n = 6, P = 0.04; protein: n = 6, P = 0.000) of ESCs, which were consistent with Cell Counting Kit-8(CCK-8) results (day 0: NC: 0.29 ± 0.04, 120 μg/mL EVs: 0.28 ± 0.04; day 1: NC: 0.42 ± 0.08, 120 μg/mL EVs: 0.32 ± 0.01; day 2: NC: 0.64 ± 0.07, 120 μg/mL EVs: 0.50 ± 0.05, P = 0.000; day 3: NC: 0.82 ± 0.09, 120 μg/mL EVs: 0.65 ± 0.07, P = 0.000; day 4: NC: 0.95 ± 0.11, 120 μg/mL EVs: 0.76 ± 0.07, P = 0.012; n = 6) and Transwell experiments (n = 6, P = 0.000). In addition, the expression of SF-1 (encoded by NR5A1; mRNA: n = 6, P = 0.000; protein: n = 6, P = 0.000), ERβ (encoded by ESR2; mRNA: n = 6, P = 0.000; protein: n = 6, P = 0.000), and aromatase (encoded by CYP19A1; mRNA: n = 6, P = 0.04; protein: n = 6, P = 0.000) in ESCs decreased significantly.

**Conclusion:**

Taken together, the results show that 120 μg/mL EVs derived from UC-MSCs can effectively inhibit the proliferation and invasion of ESCs, as well as their expression of SF-1, ERβ and aromatase, and thus may lead to the alleviation of endometriosis.

## Introduction

Endometriosis is an estrogen-dependent chronic inflammatory disorder (1–3), and it is generally believed that there is an estrogen-rich environment in patients with endometriosis. Estrogen regulates the growth of endometrial tissue through estrogen receptors. Therefore, the level of estrogen synthesis and the expression of estrogen receptors are important indicators of endometriosis. Ectopic stromal cells (ESCs) collected from ovarian endometriomas have an abnormally high expression of estrogen receptor β (ERβ), encoded by ESR2, which mediates estrogen-driven inflammatory processes (4). In addition, steroidogenic factor-1 (SF-1; encoded by NR5A1) and aromatase (encoded by CYP19A1) are also overexpressed in the ESCs of patients with endometriosis. Aromatase is the key enzyme involved in estrogen synthesis, and SF-1 dominance can induce excessive estrogen production by binding to the promoter region of aromatase (4, 5). Inhibition of ERβ, SF-1, and aromatase can decrease the production and function of local estrogen, thus leading to alleviation of endometriosis. Moreover, according to Sampson’s theory, endometriosis is characterized by the abnormal presence of endometrioid tissue located outside of the uterus (6), rendering the proliferative and invasive abilities of ESCs are important indicators of endometriosis. According to previous studies, there is an increased expression of cyclin D1 and matrix metalloproteinase (MMP) 9 in the ESCs of patients with endometriosis (7–10). Therefore, cyclin D1 and MMP9 may be potential targets for endometriosis. However, in terms of the existing treatment methods, the recurrence of endometriosis is still a major problem (11), and the development of more effective and less invasive treatment strategies is urgently needed.

Mesenchymal stem cells (MSCs) are ideal candidates for regenerative medicine (12, 13). These cells may be defined as multipotent cells capable of self-renewal that can give rise to a number of differentiated cell types (12). In recent years, the number of studies on endometriosis and MSCs has been increasing gradually (13). However, MSCs have their own shortcomings (14, 15), such as being too large to circulate through capillaries and their numbers rapidly decline after transplantation. Moreover, there is a certain risk of teratogenesis. MSCs play a role through paracrine signaling after transplantation (15). Recently, researchers have begun to investigate the use of MSCs-derived EVs as a possible therapeutic approach to retain the benefits and avoid the disadvantages of stem cell transplantation.

Extracellular vesicles (EVs) are secreted by cells and can mediate intercellular communication through transport proteins, RNAs, and lipids. EVs are often used as carriers or biomarkers for treatment because of their stability (16, 17). Existing targeted-drugs typically exhibit poor solubility or rapid inactivation in cells, and EVs present an attractive solution to circumvent these problems (18). Although there are many sources of EVs, human umbilical cord mesenchymal stem cells (UC-MSCs) are considered superior to other stems cells, because MSCs produce large quantities of EVs, and these EVs exhibit therapeutic effects and immunosuppressive activity in animal disease models. For example, Weijie Yang et al. found that MSCs-derived EVs can effectively improve the fertility of female mice by carrying functional microRNAs (19). Moreover, MSCs are typically extracted from the bone marrow, umbilical cord, cord blood, fat, and other accessible tissues. Among them, UC-MSCs are advantageous in that there are minimal ethical considerations and they are widely available, easily extracted, abundant, and have low immunogenicity (19). Thus, UC-MSCs are considered the optimal choice to explore the clinical application of stem cells and EVs. However, there are no studies on the relationship between MSCs-derived EVs and ESCs from endometriosis patients. Therefore, the aim of the present study was to investigate the therapeutic effects of MSCs-derived EVs on endometriosis. To achieve this, we cultured primary UC-MSCs, extracted EVs by ultracentrifugation, and added them to ESCs to observe changes in proliferation, invasion, and estrogen biosynthesis, and to explore the therapeutic effect of UC-MSCs-derived EVs on endometriosis. The findings of this study will provide novel insight into the clinical application of UC-MSCs, allowing for new treatment strategies for endometriosis.

## Materials and Methods

### Primary Culture of Stem Cells

The umbilical cord samples from cesarean section were collected under sterile conditions, cleaned with phosphate buffered saline (PBS), and cut into 1 mm^3^ pieces. Then, the tissue blocks were placed into culture bottles at a distance of 0.5 cm. After slowly adding MSCs’ medium (7501, Sciencell, USA), a T75 culture flask (430641, Corning, USA) was placed in an incubator at 37°C. The cells gradually emerged after 5-7 days. The medium was changed every 3-5 days. When the fusion degree was 70-80%, the cells were digested and passaged with 0.5% trypsin. After passaging to the fifth generation, the cells were identified and subsequently tested. Sample collection was approved by the ethics committee of Peking University First Hospital (No. 2020 [279]), and each patient signed an informed consent form.

### Culture of ESCs

The specimens were obtained from six women aged 23–40 years who had undergone hysterectomy by hysteroscopy combined with laparoscopy for endometriosis. All patients met the following criteria: regular menstrual history, no hormonal medication for three months, and no other endocrine, immune, or metabolic diseases. We mainly referred to the protocol of a previous study (13). In brief, specimens of ectopic endometrium were collected in a sterile environment. After washing with PBS, the tissue was cut into pieces and placed into a digestive solution [1 mg/mL collagenase (Sigma, St. Louis, Mo, USA) and 0.04 mg/mL nuclease (Sigma, St. Louis, Mo, USA)] for 60 min at 37°C. The supernatant was collected and centrifuged. Then, the pellet was resuspended in 5 mL Hank’s Balanced Salt solution. After filtration, the liquid was centrifuged again, and the sediment containing ESCs was resuspended in medium. The medium contained 10% FBS (GIBCO/BRL, PL16004, Grand Island, NY, USA), 89% Dulbecco’s modified Eagle’s medium (DMEM/F12, HyClone, Logan, UT, USA), and 1% antibiotic/antimycotic (GIBCO/BRL, Grand Island, NY, USA, 15240062). The cells were incubated in a 5% CO_2_ incubator at 37°C. The medium was changed after 24 h. The sample collection was approved by the ethics committee of Peking University First Hospital (No. 2020 [279]), and each patient signed an informed consent form.

### Identification of the Differentiation Potential of UC-MSCs

To identify the differentiation potential of the MSCs, we examined the adipogenic and osteogenic differentiation of the cells. First, to identify the adipogenic capacity of the MSCs, we used an Oil Red O staining kit (Sciencell, Oil Red Straining, No. 0843, USA). The cells were inoculated into a 6-well plate at a concentration of 10^5^ cells/well. After that, the cells in the experimental group were added to adipogenic differentiation medium (MSCs Adipogenic Differentiation Medium, Sciencell, 7541, USA), whereas cells in the control group were cultured in conventional stem cell culture medium (7501, Sciencell, USA). After 2 weeks of adipogenic induction, we performed Oil Red O staining. Cells were washed with PBS and fixed for 15 min with a fixative solution provided by the manufacturer. Then, cells were washed three times with diH_2_O to remove the fixative solution. After adding the Oil Red O staining solution, the cells were fixed at room temperature for 15 min and washed with diH_2_O five times. Finally, we observed the cells and captured the images under a microscope.

To test the osteogenic ability of MSCs, we used an Alizarin Red S staining kit (Sciencell, Alizarin Red S staining kit, No. 0223, USA). MSCs were seeded in 6-well plates at 10^5^ cells/well, and then cultured in osteoblast medium (50 mL osteoblast MSCM + 5 mL FBS + 0.5 mL double antibody + 0.5 mL MSCGS; Sciencell, MSC osteogenic differentiation medium, 7531, USA) after growth and fusion. The control group was cultured in conventional medium (7501, Sciencell, USA). After two weeks of osteogenic induction, alizarin s staining was performed, and 2% Alizarin Red S (1 mL) was added to each well and incubated for 20-30 min at room temperature, before the cells were washed five times with diH_2_O. Then, 1 mL/well diH_2_O was added to prevent the cells from drying. Images were taken under a microscope.

### Extraction of EVs

We used ultracentrifugation to obtain EVs. Medium for EVs collection were harvested after one day in culture. First, to remove the cell debris, the MSC medium was collected and centrifuged at 500 × *g* for 10 min. Next, the supernatant was centrifuged at 16,000 × *g* for 20 min to remove apoptotic bodies. Then, 150,000 × *g* ultracentrifugation was performed for 70 min and the precipitate was resuspended in PBS. After that, 150,000 × *g* ultracentrifugation was carried out for another 70 min. Bacteria and impurities were then removed at 10,000 × *g* for 10 min. The EVs were quantified using a Nanodrop. During the whole experiment, in order to ensure the freshness and effect of exosomes, exosomes were prepared on the spot.

### Electron Microscopy

To observe the morphology of EVs by electron microscopy (14, 15), we collected the EVs, added 4% paraformaldehyde, stored them at 4°C, and sent them to be viewed within a week. The samples were sent to the electron microscope laboratory of the Peking University Health Science Center. In brief, 10 μL of the sample was added to the carbon-coated copper mesh for 90 s. The excess liquid was removed with filter paper. Then, 10 μL of uranyl acetate staining solution was added to the copper mesh and incubated in the dark for 30 s. The excess liquid was removed with filter paper and the sample was air-dried. Finally, the sample was observed and photographed under a transmission electron microscope.

### Nanoparticle Tracking Analysis (NTA)

NTA technology is an established exosome characterization method. This approach involves the tracking and analysis of the Brownian motion of each particle, and calculates the hydrodynamic diameter and concentration of nanoparticles. In brief, the EVs were collected according to the above ultracentrifugation method and stored at 4°C for no longer than a week. The concentration and particle size of the EVs were detected by Wayen Biotechnology (Shanghai, China).

### Characterization of Stem Cells and EVs by Flow Cytometry

After digestion with trypsin, the cells were centrifuged at 1000 rpm for 10 min and washed twice with PBS. The cells were resuspended in PBS, centrifuged for 6 min, and the supernatant was discarded. One microliter of antibody/tube was added to the cells. These antibodies included positive markers (anti-CD90, 328117, PerCP/Cy5.5 mouse IgG1, κ isotype Ctrl, 400149; anti-CD44, 338803, FITC mouse IgG1, κ isotype Ctrl (FC), 400109; anti-CD29, 303003, PE mouse IgG1, κ isotype Ctrl (FC), 400113; anti-CD105, 800507, PerCP/Cy5.5 mouse IgG1, κ isotype Ctrl, 400149; BioLegend, San Diego, CA, USA) and negative markers (anti-CD19, 302207, PE mouse IgG1, κ isotype Ctrl (FC), 400113; HLA-DR, 307605, PE mouse IgG2a, κ isotype Ctrl (FC), 400113; BioLegend, San Diego, CA, USA). After incubation at 4 °C for 30 min, 1 mL PBS was added to the tubes, which were then centrifuged at 2000 rpm for 6 min. This step was repeated twice. Finally, the cells were resuspended in 0.5 mL PBS and subjected to flow cytometric analysis. The identification procedure of exosome surface markers were similarly performed. Among them, anti-CD63 (312005, PE mouse IgG1, κ isotype Ctrl (FC), 400113) and anti-CD81 (349505, PE mouse IgG1, κ isotype Ctrl (FC), 400113) were purchased from the BioLegend (San Diego, CA, USA).

### Real-Time Quantitative PCR (qRT-PCR)

To detect the mRNA expression of ESCs stimulated with EVs, the total RNA was extracted. In brief, ESCs in the exponential growth period were placed on ice and Trizol (Invitrogen, Carlsbad, CA, USA) was added. After 5–10 min, chloroform was added and mixed thoroughly; samples were incubated at 4°C for 10–15 min. Samples were then centrifuged at 12,000 rpm at 4°C for 15 min. The resulting supernatant was then combined with an equal volume of isopropanol to precipitate the RNA, and samples were incubated at 4°C for 10 min. After centrifugation at 12,000 rpm for 10 min, white precipitates were observed on the side wall of the EP tube. After washing with 75% alcohol, the organic matter in the precipitate was separated. RNA concentration was determined using a Nanodrop, and used immediately or stored at –80°C. Transcript one-step gDNA removal and cDNA synthesis Supermix (AT311-03, TransGen Biotech, China) were used in the reverse transcription experiment. In the qRT-PCR experiment, we used an ABI power SYBR Green gene expression system (Applied Biosystems, USA). The target genes were MMP9, cyclin D1, NR5A1 (SF-1), CYP19A1 (aromatase), and ESR2 (ERβ). The internal reference gene was β-actin. The primer sequences were as follows: β-actin forward, 5’-CACGGCTGCTTCCAGCTC-3’, reverse, 5’-CACAGGACTCCATGCCCAG-3’; MMP9 forward, 5’-AGACCTGGGCAGATTCCAAAC-3’, reverse, 5’-CGGCAAGTCTTCCGAGTAGT-3’; cyclin D1 forward, 5’-CAATGACCCCGCACGATTTC-3’, reverse, 5’-CATGGAGGGCGGATTGGAA-3’; CYP19A1 forward, 5’-CACATCCTCAATACCAGGTCC-3’, reverse, 5’-CAGAGATCCAGACTCGCATG-3’; NR5A1 forward, 5’-ATTGAAGTCACTAGAGATGGCCT-3’, reverse, 5’- CTTATCATCTTGCCCTTGAGTGG-3’; and ESR2 forward, 5’-TGCTCCCACTTAGAGGTCAC-3’, reverse, 5’-GAAAAGATCA CAAGCGACTTAACG -3’.

### Cell Counting Kit-8 (CCK8) Assay

We used the CCK-8 assay (Dojindo Molecular Technologies, XiongBen, Japan) to assess the proliferative ability of the ESCs. First, when ESCs reached 70-80% confluence, they were digested and seeded in 96-well plates in 100 μL of medium at a concentration of 3000 cells/well. The culture medium of the experimental group contained 120 μg/mL EVs, and an equal amount of PBS was added to the control group. The cells were then cultured in an incubator at 37°C and 5% CO_2_. On day 0, 1, 2, 3, and 4, 10 μL of CCK-8 reagent was added to each well, as per the manufacturer’s instructions. ESCs were incubated with the reagent for 1.5 h, before the absorption was determined at 450 nm. Six samples were tested, and each sample was repeated six times at each time point. OD values were expressed as mean ± standard deviation, and an independent sample t-test was used between two groups of samples.

### Trypan Blue Assay

We assessed the living rate of ESCs using Trypan blue staining. In short, when ESCs reached 70-80% fusion, they were digested by trypsin and prepared into cell suspensions. When the cell suspension was mixed with 0.4% trypan blue solution at a ratio of 9:1, the number of living and dead cells was counted within 3 minutes. Among them, the rate of living cells (%) = the total number of living cells/(total number of living cells + total number of dead cells) x 100%.

### Matrigel Invasion Assay

A matrigel invasion assay was used to detect the invasive ability of ESCs. First, matrigel (BD company, Franklin Lakes, NJ, USA) was diluted 1:8 and added to the upper chamber of the Transwell plate. The matrigel condensed into a gel after 30 min in an incubator at 37°C. Next, the ESCs were digested by trypsin, centrifuged, and washed twice with PBS. The experimental group was subjected to serum-free medium containing 120 μg/mL EVs, whereas the control group medium was prepared by adding the same volume of PBS. We added the cell suspension into the upper chamber (10,000 cells/well). Then, 600 μL of medium containing 20% serum was added to the lower chambers, carefully to avoid bubbles. The cells were cultured for 48 h under conventional conditions. Then, the medium was discarded and cells were washed twice with PBS, and the upper cells were gently removed with a cotton swab. The samples were fixed with 4% paraformaldehyde for 30 min, stained with 0.1% crystal violet for 20 min, and washed with deionized water twice. Finally, the samples were viewed and the number of cells was counted under a microscope. Six samples were selected, and five random fields-of-view were selected for each sample.

### Western Blotting

Western blot analysis was used to detect the expression of various proteins in the cells. Briefly, the cells were lysed and the protein quantified through a bicinchoninic acid assay. In general, 20 μg of total protein was injected into each well. After electrophoresis and electro-conversion, the membranes were sealed with 5% skim milk at room temperature for 1 h. Next, the membranes were incubated overnight with antibodies. The following antibodies were used: anti-MMP9 (dilution, 1:1000; Abcam; ab76003, Cambridge, UK); anti-cyclin D1 (dilution, 1:1000; Cell Signaling Technology; 92g2, MA, USA); anti-aromatase (dilution, 1:1000; Abcam; ab35604, Cambridge, UK); anti-SF-1 (dilution, 1:500; Abcam; ab65815, Cambridge, UK); and anti-ERβ (dilution, 1:1000; Merck Millipore; 04–824). In addition, anti-GAPDH (dilution, 1:1000; ZSGB-bio, Beijing, China) was used as an experimental control. ImageJ software (National Institutes of Health, Bethesda, USA) was used to evaluate protein expression.

### Data Analysis

All data were analyzed using SPSS 23.0 software, with P < 0.05 considered statistically significant. The Chi-square test was used for counting data. An independent sample t-test was used between two groups of samples with normal distribution, and analysis of variance was used between multiple groups of samples. The data for all samples represent at least three repeats.

## Results

### Identification of Stem Cells

**Figure 1:**
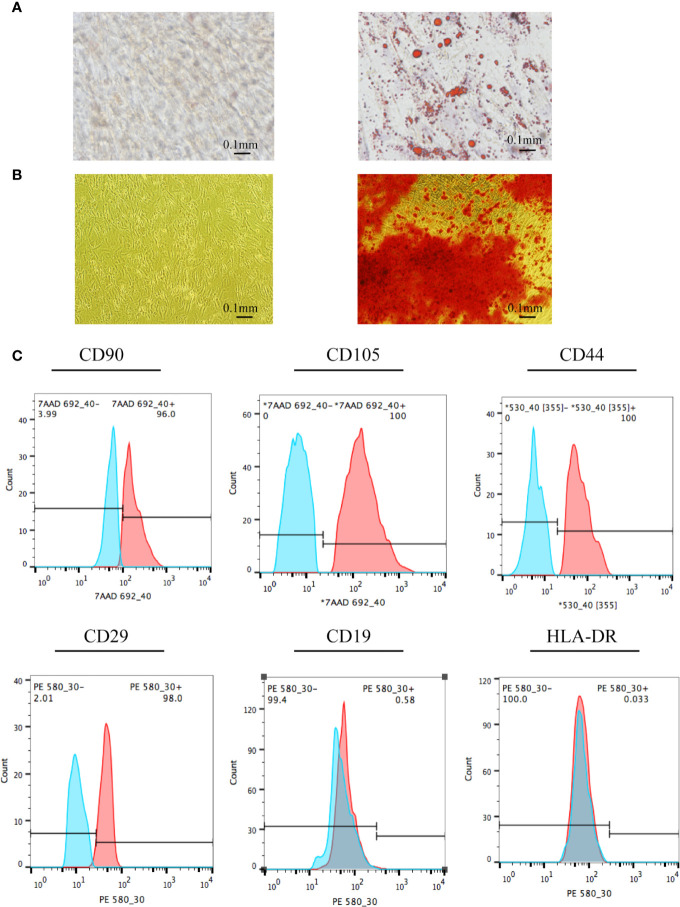
Identification of UC-MSCs. **(A)** UC-MSCs were exposed to adipogenic differentiation medium and stained by Oil Red O. Compared to cells in the control group, the red, round lipid droplets observed in UC-MSCs treated with adipogenic differentiation medium confirms their differentiation into adipocytes. **(B)** UC-MSCs were cultured with osteoblast differentiation medium and stained by Alizarin Red S. As evidenced by the red round droplet in the righthand image, UC-MSCs could differentiate into osteocytes. **(C)** Stem cell phenotypes were identified by flow cytometry. Positive indicators (CD90, CD105, CD44, and CD29) showed that the cells to be tested were stem cells rather than ordinary cells. In addition, negative markers (CD19 and HLA-DR) showed that the cells to be tested were mesenchymal stem cells rather than hematopoietic stem cells. All experiments were repeated three times.

Stem cell identification was the basis for confirming the origin of the EVs. Compared to the control group (conventional stem cell culture medium), the cells in the experimental group exposed to adipogenic differentiation medium had multiple red, round lipid droplets (**Figure 1A**), indicating that adipocyte differentiation was successful. The UC-MSCs could also differentiate into osteocytes, as characterized by the red substance observed (**Figure 1B**). Furthermore, the surface markers of UC-MSCs were identified *via* flow cytometry. Positive markers were used to identify the cultured cells as stem cells rather than normal cells, and negative markers were used to differentiate hematopoietic stem cells from MSCs. The positive rates of the positive indicators CD90, CD105, CD44, and CD29 were 96.00%, 100.00%, 100.00%, and 98.00%, respectively (**Figure 1C**). The positive rates of the negative indicators CD19 and HLA-DR were 0.58% and 0.03%, respectively. The above results confirmed that the sample cells were UC-MSCs.

### Identification of EVs

**Figure 2:**
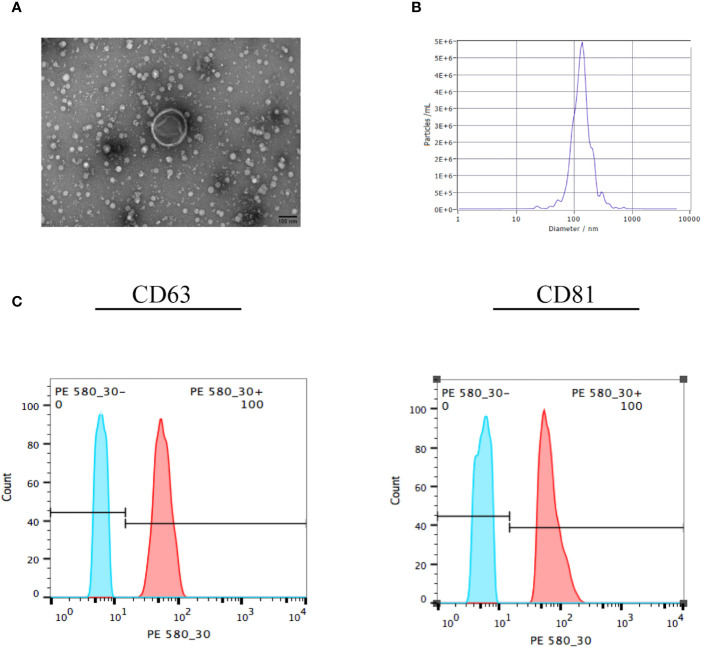
Identification of EVs. **(A)** The EVs were collected and observed by electron microscopy. The exosome in the visual field was approximately 100 nm, with a saucer-like structure and clear membrane. **(B)** The EVs were identified by nanoparticle tracking analysis, which showed that the median size of the vesicle was 130.10 nm. **(C)** The EVs were collected and surface markers were identified by flow cytometry. The positive rates of CD63 and CD81 were 100%, which meant that the vesicles were EVs. All experiments were repeated three times.

Confirming the identification of EVs before their addition to ESCs was essential. There are many methods to identify EVs, among which electron microscopy is the most reliable. We used ultracentrifugation to extract the vesicles secreted by UC-MSCs, which exhibited a saucer-like structure with a clear membrane, approximately 100 nm in length (**Figure 2A**). This was consistent with the expected structure. The results for NTA (**Figure 2B**) showed that the sample concentration was 5.3 E+7 particles/mL. The median size of the vesicles was 130.10 nm, which was consistent with the characteristics of EVs. In addition, we used flow cytometry to detect exosome surface markers (**Figure 2C**). We found that both the positive rates of CD63 and CD81 were 100%, which meant that the extracted vesicles were EVs.

### EVs Can Inhibit the Proliferation and Invasion of ESCs

**Figure 3:**
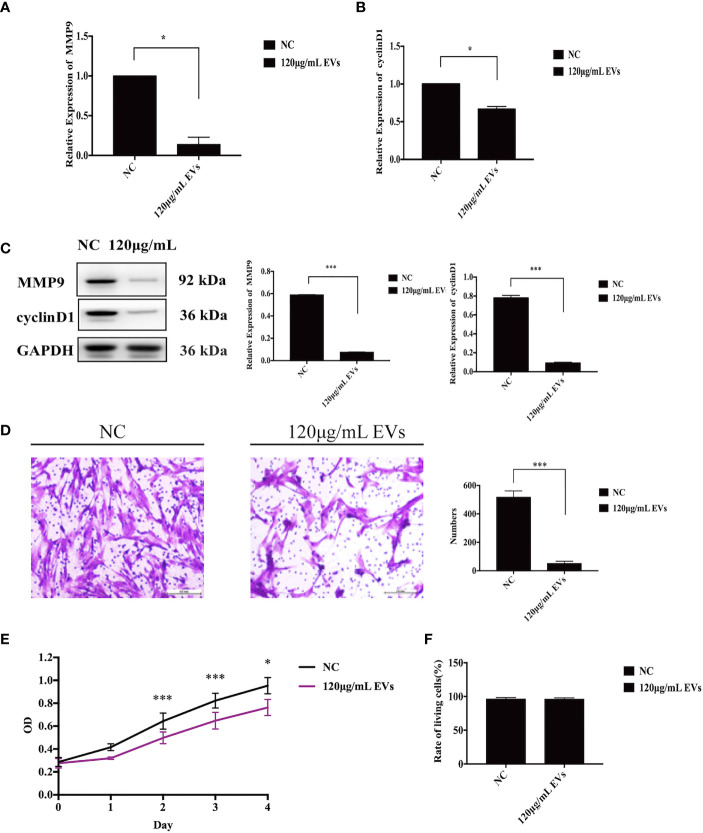
EVs inhibit ESC proliferation and invasion. **(A)** ESCs were collected for qRT-PCR to detect to mRNA expression of MMP9 (n = 6, P = 0.04), after stimulation with EVs. **(B)** After stimulation with EVs, ESCs were collected for qRT-PCR to identify the mRNA expression of cyclin D1 (n = 6, P = 0.02). **(C)** ESCs were collected for western blotting analysis using the anti-MMP9 and anti-cyclin D1 (n = 6, P = 0.000 each). **(D)** A matrigel invasion assay was used to detect the invasive ability of ESCs. The invasive ability of ESCs exposed to 120 μg/mL EVs showed a 90% reduction (n = 6, P = 0.000). **(E)** CCK-8 assay was used to assess the proliferative ability of ESCs between days 0–4. All experiments were repeated three times. **(F)** Trypan blue staining was used to assesse the living rate of ESCs. *P < 0.05, ***P < 0.001 *vs.* individual controls. OD values in CCK-8 assays and cell numbers in Transwell assays were expressed as mean ± standard deviation. Independent sample t-test was used between two groups of samples.

Changes in the proliferative and invasive abilities of ESCs following exosome stimulation are important considerations when examining the therapeutic potential of EVs on endometriosis. Compared with the control group, mRNA levels of MMP9 and cyclin D1 decreased by 86% (n = 6, P = 0.04, **Figure 3A**) and 33% (n = 6, P= 0.02, **Figure 3B**) with 120 μg/mL EV treatment, respectively. Likewise, the protein levels of MMP9 and cyclin D1 decreased with 120 μg/mL EV treatment (n = 6, P = 0.000 each, **Figure 3C**). Compared to cells in the control group, the invasive ability of ESCs exposed to 120 μg/mL EV showed a 90% reduction (n = 6, P = 0.000, **Figure 3D**). Furthermore, we assessed cell proliferation over days 0 – 4. The OD values measured at 450 nm, which could indirectly reflect the number of living cells, showed that the proliferative capacity of ESCs stimulated with 120 μg/mL EVs significantly decreased (day 0: NC: 0.29 ± 0.04, 120 μg/mL EVs: 0.28 ± 0.04; day 1: NC: 0.42 ± 0.08, 120 μg/mL EVs: 0.32 ± 0.01; day 2: NC: 0.64 ± 0.07, 120 μg/mL EVs: 0.50 ± 0.05, P = 0.000; day 3: NC: 0.82 ± 0.09, 120 μg/mL EVs: 0.65 ± 0.07, P = 0.000; day 4: NC: 0.95 ± 0.11, 120 μg/mL EVs: 0.76 ± 0.07, P = 0.012; n = 6, **Figure 3E**). In Trypan blue staining (**Figure 3F**), there was no significant difference in the rate of living cells between the two groups (NC: 96.03% ± 2.31%; 120 μg/mL EVs: 95.99% ± 2.03%, n = 6, P > 0.05). In summary, 120 μg/mL EVs could inhibit ESCs proliferation and invasion.

### EVs Inhibit the Expression of SF-1, Aromatase, and ERβ of ESCs

**Figure 4:**
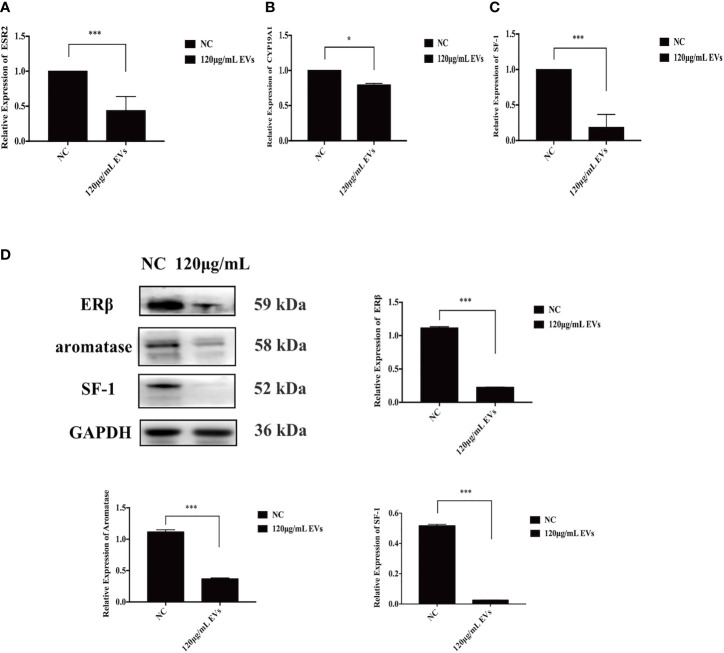
EVs inhibit expression of SF-1, aromatase, and ERβ in ESCs. EVs have a certain inhibitory effect on the expression of estrogen-related genes in ESCs. **(A–C)** ESCs were collected for qRT-PCR. At the mRNA level, compared with the control group, the expression of ESR2, CYP19A1, and SF-1 decreased by 81.67% (n = 6, P = 0.000), 21.50% (n = 6, P = 0.04), and 51.64% (n = 6, P = 0.000), respectively. **(D)** ESCs were collected for western blotting analysis using anti-ERβ, anti-SF-1, and anti-aromatase antibodies. The protein expression of ERβ, SF-1, and aromatase decreased (n = 6, P = 0.000 for all proteins). All experiments were repeated three times.*P < 0.05, ***P < 0.001 *vs.* individual controls. Data in qRT-PCR and wWestern blotting were shown as mean ± standard deviation. Independent sample t-test was used between two groups of samples.

To assess the effects of EVs on the expression of estrogen-related proteins (SF-1, aromatase, and ERβ) in ESCs, qRT-PCR and western blotting were used. At the mRNA level, compared to ESCs in the control group, the expression of ESR2, CYP19A1, and SF-1 decreased by 56.14% (n = 6, P = 0.000, **Figure 4A**), 21.50% (n = 6, P = 0.04, **Figure 4B**), and 81.67%, respectively, when exposed to 120 μg/mL EVs (n = 6, P = 0.000, **Figure 4C**). Similarly, western blotting analysis showed that the expression of SF-1, aromatase, and ERβ decreased (n = 6, P = 0.000 for all proteins, **Figure 4D**). In conclusion, 120 μg/mL EVs reduced the expression of estrogen-related genes in ESCs.

## Discussion

Overall, this study showed that 120 μg/mL EVs significantly decreased ESCs’ proliferation and invasion, and significantly decreased the expression of SF-1, ERβ, and aromatase. The findings of this study provide novel insight into the potential clinical utility of UC-MSCs-derived EVs as an effective treatment option for endometriosis.

We innovatively hypothesized that EVs derived from UC-MSCs could be used to treat endometriosis. At present, there have been preliminary investigations into the relationship between EVs and endometriosis. Various endometriosis exosome biomarkers have been identified through sequencing (20–22). These biomarkers include long noncoding RNAs, microRNAs, and mRNAs, identified from the EVs of stromal cells, peritoneal fluid (21), and serum (22). The therapeutic roles of EVs have also been explored. For example, Harp D (23) used the ESCs EVs to explore the effect of enhancing angiogenesis *in vitro*. Wu et al. (24) found that miR-214 secreted by ESCs could inhibit endometriosis-related fibrosis. Zhang L (25). suggested that miR-22-3p derived from peritoneal macrophages could promote the proliferation, migration and invasion of endometrial stromal cells by regulating the Sirt1/NF-κB signaling pathway. However, this type of research has certain limitations, including that these studies focused mostly on disease etiology, and that exosome production by these cells, such as ESCs, is relatively low. That is to say, clinical translation research is relatively difficult. Studies have shown that MSCs are the cells that produce the most EVs (11). In the present study, we focused on the therapeutic application of EVs, which could benefit future clinical transformation. UC-MSCs have the advantages of stem cell transplantation, such as safety, non-toxic properties, and the potential for large-scale production. UC-MSCs-derived EVs avoid the disadvantages of stem cells; for example, the risk of teratogenesis is lower and they pass more easily through capillaries. Therefore, compared to previous studies, the overall experimental design is of great significance to the treatment of endometriosis.

We hypothesized that the EVs secreted by UC-MSCs can be absorbed by ESCs and inhibit proliferation and invasion of ESCs, which were confirmed by CCK-8 and Transwell assays, respectively. Researchers believe that inhibiting the production of local estrogen can alleviate the symptoms of endometriosis (26). Aromatase is the key enzyme in estrogen synthesis, and SF-1 can bind to all estrogen synthetases (including aromatase). The results showed that, compared to ESCs in the control group, the expression of aromatase and SF-1 in cells in the experimental group was significantly decreased. Moreover, we also observed a significant decrease in ERβ expression at the mRNA and protein levels. Therefore, it is reasonable to speculate that EVs at this concentration can inhibit the production and function of estrogen, and reduce local estrogen levels in ectopic lesions, which is of great significance in terms of alleviating and treating endometriosis.

We found that the effect of EVs was concentration-dependent. Medium with 30 and 60 μg/mL EVs did not cause significant changes in ESCs. Only when the concentration reached 120 μg/mL, did the proliferation, invasion, and expression of estrogen-related proteins decrease significantly. This is of importance when selecting the optimal dose for future clinical application. What’s more, in Trypan blue staining, no obvious difference was found. We believed that EVs at this concentration have little effect on cell death.

Study limitations include the lack of relevant animal experiments and the lack of in-depth functional studies, which are to be addressed in future studies undertaken by our research group. This study provides a theoretical basis for the treatment of UC-MSCs-derived EVs in endometriosis. At the same time, the effect of EVs on eutopic stromal cells needs to be further explored. However, the utility and efficacy of these EVs depend on many parameters (9, 27, 28), such as the storage conditions of EVs, amplification methods, administration forms, and individual differences, which are research priorities in the future.

## Conclusions

EVs derived from UC-MSCs can effectively inhibit the proliferation and invasion of ESCs, as well as the expression of SF-1, ERβ, and aromatase, thereby restricting the growth, invasion, and estrogen levels of ectopic lesions, thus leading to the alleviation of endometriosis.

## Data Availability Statement

The raw data supporting the conclusions of this article will be made available by the authors, without undue reservation.

## Ethics Statement

The studies involving human participants were reviewed and approved by the ethics committee of Peking University First Hospital (No. 2020 [279]). The patients/participants provided their written informed consent to participate in this study.

## Author Contributions

Design and obtaining funding, QX. Drafting of the manuscript, XQ-W. Collection of samples, XL. Analysis and interpretation of data, PL-W, CZ, and JW-Z. Critical revision of the manuscript for important intellectual content, YL, YF-Z, and QX. All authors contributed to the article and approved the submitted version.

## Funding

This work was supported by the National Key R&D Program of China [grant number 2017YFC1001203].

## Conflict of Interest

The authors declare that the research was conducted in the absence of any commercial or financial relationships that could be construed as a potential conflict of interest.

## Publisher’s Note

All claims expressed in this article are solely those of the authors and do not necessarily represent those of their affiliated organizations, or those of the publisher, the editors and the reviewers. Any product that may be evaluated in this article, or claim that may be made by its manufacturer, is not guaranteed or endorsed by the publisher.
